# Is general inpatient obstetrics and gynaecology evidence-based? A survey of practice with critical review of methodological issues

**DOI:** 10.1186/1472-6874-6-5

**Published:** 2006-03-10

**Authors:** Aamir T Khan, M Nauman Mehr, Anne-Marie Gaynor, Malcolm Bowcock, Khalid S Khan

**Affiliations:** 1 Department of Obstetrics and Gynaecology, Birmingham Womens NHS Trust, UK; 2 Department of Practice Development, Birmingham Womens NHS Trust, UK; 3 Clinical Governance and Audit Department, Birmingham Womens NHS Trust, UK

## Abstract

**Background:**

To examine the rates of evidence-supported care provided in an obstetrics-gynaecology unit.

**Methods:**

The main diagnosis-intervention set was established for a sample of 325 consecutive inpatient admissions in 1998–99 in a prospective study in a UK tertiary care centre. A comprehensive literature search was conducted to obtain the evidence supporting the intervention categorised according to the following hierarchy: Grade A, care supported by evidence from randomised controlled trials; Grade B, care supported by evidence from controlled observational studies and convincing non-randomised evidence; and Grade C, care without substantial research evidence.

**Results:**

Of the 325 admissions, in 135 (42%) the quality of care was based on Grade A evidence, in 157 (48%) it was based on Grade B evidence, and in 33 (10%) it was based on Grade C evidence. The patterns of care were not different amongst patients sampled in 1998 and 1999.

**Conclusion:**

A significant majority (90%) of obstetric and gynaecological care was found to be supported by substantial research evidence.

## Background

The practice of medicine is underpinned by clinical experience, intuitive clinical reasoning and applied medical research. In recent times, there has been a shift towards evidence-based medicine (EBM) defined as the conscientious, explicit and judicious use of contemporary best research evidence in making decisions about the care of individual patients [1]. To what extent is this concept being applied in clinical practice? This approach gained significant momentum in the 1990's. In thoracic surgery 78% of patient care [2], in general medicine 82% [3], in general surgery 95% [4], in paediatric medicine 75% [5], in ophthalmology 77% [6], and in anaesthesia 97% [7] of care is purported to be evidence-based. No such evidence exists in obstetric and gynaecological care. Such evaluations are fraught with methodological difficulties, which to our knowledge have not been explored in the literature. We thus assessed the extent of evidence-based care in an inpatient obstetrics-gynaecological unit and generated examples of potential pitfalls in research on evidence-based practice.

## Methods

All inpatient admissions at a tertiary care centre in 1998 and 1999 during the first week of October were reviewed. Our approach replicated previous study designs [2-7]. We selected cases for analysis where an active diagnosis was accompanied by an active intervention. This meant that chronic background conditions requiring ambulatory treatment that were not the reason for inpatient admission were not considered. The main diagnosis-intervention sets were coded using the International Statistical Classification of Diseases and Related Health Problems (ICD-10) [8]. There were 298 and 290 consecutive cases during the two sampling periods, of which 85 and 67 were excluded due to admissions without interventions (admissions requiring monitoring or observation only, e.g. in cases of threatened perterm labour that spontaneously subsides) and 45 and 66 were excluded due to generation of confusing sets using the ICD-10 (see discussion – Table 2) in 1998 and 1999 respectively. This left 168 and 157 admissions for analysis in 1998 and 1999 respectively. To determine the codes we examined case notes as our previous research had shown a high degree of inaccuracy in electronic data [9]. Any secondary diagnoses or interventions were excluded from analysis. The data extraction was performed by three of us (AMG, TB and PC).

**Table 1:** Rates of evidence-based inpatient management in obstetrics and gynaecology

Grades of evidence*	**Total**		**1998+**		**1999+**	
			
	**n**	**%**	**n**	**%**	**n**	**%**
Grade A	135	42	69	41	66	42
Grade B	157	48	79	47	78	50
Grade C	33	10	20	12	13	8

**Total no. of admissions**	**325**	**100**	**168**	**100**	**157**	**100**

n = number of patients

* See methods for details

+ No difference between grades for the two sampling periods (p = 0.9)

**Table 2:** Potential pitfalls in research on rates of evidence-based practice

**Potential pitfalls and explanation of methodological issues**
**Grading of interventions**
The interventions themselves do not carry a grade, it is their application in the appropriate clinical circumstances that earns them the relevant evidence grade. For example, a hysterectomy for menorrhagia may be appropriately graded A only when less invasive options have been exhausted. This problem may not be dealt with by pairing up interventions with diagnoses without regard for previous history of the problem.
**Selection of main diagnosis-intervention set**
By narrowing down to one main set other aspects of care that might be important might be excluded. An alternate approach would be to develop care-pathways based on evidence and study compliance with pathway as a measure of evidence-based practice.
**Multi-faceted interventions**
Some interventions are a composite of several aspects of care, e.g. management of labour consists of amniotomy, augmentation, support, etc. Each aspect of care may be evidence based but it may be difficult to provide a single grade to the composite intervention.
**Coding of diagnosis-intervention sets**
Coding using the International Statistical Classification of Diseases and Related Health Problems (ICD-10) (WHO, 1992) can produce confusing sets, e.g. vertex presentation and normal delivery. Here the delivery is an outcome not an intervention. The intervention is care according to labour ward guideline. Sometimes such sets could be so confusing that they cannot qualify for an evidence search.
**Unit of analysis**
If admission is used as the unit of analysis instead of patient this might bias the analysis. It is possible that the same patient may be counted more than once if they are admitted on several occasions over the study period. Using short sampling periods may avoid this problem.
**Self evident interventions**
These are interventions where there are no controlled trials in support of the treatment modality but there is convincing biological or basic research evidence such that a trial would be unnecessary or unethical, e.g. caesarean section for placenta praevia. These should not be graded as Grade C.
**Cost-effectiveness of care**
Cost-effectiveness rather than effectiveness alone may determine provision of care. Some cases may be graded lower on the grounds that the marginal benefit of an intervention graded higher is not considered worthy of the additional expense involved.

Medical literature was searched and examined by two of us (NM and ATK) to determine whether or not the main diagnosis-intervention set for each admission was supported by research evidence using a systematic hierarchical approach [10]. Any disagreements between the reviewers were resolved by consensus between them or by arbitration by a third reviewer (KSK). Our search interrogated the Guidelines from the Royal College of Obstetricians and Gynaecologists [11], Guidelines from the Scottish Intercollegiate Guidelines Network [12], the National Electronic Library for Health [13], Cochrane Database [14], the Reproductive Health Library [15], Clinical Evidence [16], PubMed Medline [17], and other bibliographic data bases. Our search was guided by the principle of "theoretic saturation", i.e. we stopped search as soon as relevant graded evidence was identified. This approach has wide acceptance in the research community [18].

Key references from the search were used to assign each diagnosis-intervention set to one of the following three categories of evidence: Grade A, care supported by evidence from randomised controlled trials (RCTs); Grade B, care supported by evidence from controlled observational studies as well as conditions where there were no controlled data in support of the interventions but there were convincing biological or basic research evidence such that controlled trials would be unnecessary or unethical; Grade C, care without substantial research evidence. Grade A evidence was presumed to be of the highest quality. Grade B evidence including non-randomised prospective cohort studies and large retrospective comparative studies, was second best. Where Grade B was based on inherent validity of interventions we sought consensus from two to three consultants. Both Grade A and B were considered substantial supportive evidence. We computed rates of evidence-based practice separately for the two sampling periods and sought for examples of methodological pitfalls, which we discuss while examining the validity of our findings.

## Results

Of the complete list of 325 consecutive inpatient admissions (Table 1) the care provided in 135 (42%) was supported by Grade A evidence, in 157 (48%) the care was supported by Grade B evidence, and in 33 (10%) the care was supported by Grade C evidence. Of the 157 cases graded B, there were 23 (15%) based on convincing biological or basic research evidence when trials would be unnecessary or unethical. There were no significant differences in pattern of care amongst the patients sampled in 1998 and 1999.

## Discussion

Medical practice has been criticised as not being based on solid evidence [19]. No published studies have examined the extent of EBM in general obstetric and gynaecological practice. Our study showed that the majority (90%) of the care among general obstetric and gynaecological inpatient admissions was well supported by research evidence.

The validity of our findings depends on the methodological robustness of research designs used to evaluate evidence-based practice [2-7]. The strength of our study is that we evaluated a consecutive series and examined actual case notes. This reduces the risk of bias due to mis-classification of diagnosis-intervention sets. Our searches were extensive and the grades of evidence were extracted largely from the guidelines of bodies responsible for practice in the UK. The methodological deficiencies that must be understood in order to realistically interpret our findings are summarised in Table 2, where we have identified possible pitfalls in research on rates of evidence-based practice. Moreover, the generalisability of our findings may be limited as these are based on a study performed in one hospital in 1998–99. Our study may be considered 'not particularly up to date' by some critics. However, profound changes seldom take place quickly in healthcare as practitioners have difficulty finding, assessing, interpreting, and applying evidence. Another contributory factor is the slow progress in accumulation of strong evidence over the last half decade. Hence, for the majority of the gynaecologic and obstetric conditions included in our study practice has remained unchanged. On balance, we are confident that the rates and grades summarised in our study merit consideration.

EBM requires application of knowledge of medical informatics (i.e. efficiently searching the medical literature) and clinical epidemiology (i.e. being able to critically appraise the literature) along with intuition and experience to improve decision-making for individual patients [20]. Provision of up-to-date medical information may promote the application of research evidence and may lead to improvements in healthcare [21]. A Cochrane systematic review has concluded that audit and feedback can be effective in bringing about improvements in the performance of healthcare provision, which are worthwhile [22]. The identification of potential difficulties in undertaking research on evidence-based practice may help to clarify how future assessments of EBM may be developed. Our findings may serve as a baseline for comparison. Our study and careful dissection of the approach used provides insight into how the rates of evidence-based practice may be more adequately assessed in practice.

## Authors' contributions

KSK designed the study, supervised the work, analysed and interpreted the data, and helped draft the manuscript; ATK and MNM extracted the data, transferred it to a database designed by MB, and drafted the first manuscript; MNM extracted the data and transferred it to a database designed by MB; A-MG helped design the study and coordinated the work; and MB helped design the study, developed and maintained the study database, and coordinated and supervised extraction of information from case notes and literature. All authors read and approved the final manuscript.

## Pre-publication history

The pre-publication history for this paper can be accessed here:


